# The expanding role of Tax in transcription

**DOI:** 10.1186/1742-4690-1-19

**Published:** 2004-07-30

**Authors:** Cynthia de la Fuente, Fatah Kashanchi

**Affiliations:** 1 Institute for Proteomics Technology and Application, The George Washington University, Washington, DC 20037, USA; 2 Department of Biochemistry and Molecular Biology, The George Washington University School of Medicine, Washington, DC 20037, USA; 3 The Institute for Genomic Research (TIGR), Rockville, MD 20850, USA

## Abstract

The viral transactivator of HTLV-I, Tax, has long been shown to target the earliest steps of transcription by forming quaternary complexes with sequence specific transcription factors and histone-modifying enzymes in the LTR of HTLV-I. However, a new study suggests that Tax preferentially transactivates the 21-bp repeats through CREB1 and not other bZIP proteins. The additional transactivation of Tax-responsive promoters subsequent to initiation is also presented. This result highlights a potentially novel role of Tax following TBP recruitment (*i.e. *initiation) and may expand the mechanism of Tax transactivation in promoter clearance and transcriptional elongation.

## 

Viruses have long been a source of key scientific discoveries. Historically, they have contributed to our knowledge of transcription, cell cycle, and apoptosis. To date activated transcription in higher eukaryotic cells with or without chromatin is a great area of active research and many researchers use viral activators, including herpes virus VP16, adenovirus E1A, HIV-1 Tat and HTLV-I Tax to not only understand viral, but also basic mechanisms related to host control of vital cellular machineries, including transcription. Eukaryotic transcription has five distinct phases, pre-initiation, initiation, promoter clearance, elongation and termination, and is a tightly regulated and coupled process [1]. Viral transactivators, such as Tax, have long been shown to target the earliest steps of transcription by forming quaternary complexes with sequence specific transcription factors and histone-modifying enzymes in the LTR of HTLV-I. These Tax-containing complexes allow for increased recruitment of TBP (TFIID), GTFs, and RNAP II within the core promoter region, leading to the synthesis of viral RNA. However, determination of those cellular factors important for enhanced transcriptional activity, as well as the full scope of Tax transactivation, is still not fully elucidated.

In the report by Ching *et al. *[2] the authors directly compare which HTLV-I enhancer motif is preferred by Tax. Each enhancer element (21-bp, CRE, AP1, SP1, κB, or SRE) was placed in an identical TATAA-context to generate a minimal HTLV-I promoter. Previous studies had utilized various promoters (which contain additional DNA elements) to highlight a particular enhancer element necessary for Tax transactivation. Thus, this is the first study to directly compare these elements in an identical setting. In the presence of Tax, the 21-bp repeat (also known as the viral CRE elements or TxREs) was found to be most responsive (70-fold above basal levels). The 21-bp repeat was clearly preferred by Tax, since other enhancer elements were only stimulated 10-fold or less. Previously, several studies suggested that Tax activation of the 21-bp repeats may be mediated by ATF-4 [3-5]. It was shown that Tax was able to interact with ATF-4 bound to the 21-bp repeats, enhance the binding of ATF-4 to the enhancer, and recruit CREB binding protein (CBP) to the viral promoter [5]. Recently, CREB1 and ATF-4, in addition to ATF-1 and ATF-2, were found to be present *in vivo *on the 21-bp repeats (viral CRE elements) in HTLV-I infected cells through chromatin immunoprecipitation (ChIP) assays [6]. By using dominant negative mutants of CREB1, ATF-4 (CREB2/TAXREB67), Fos, and LZIP, Ching *et al. *demonstrated that among the various bZIP proteins, CREB1 was clearly favored for Tax transactivation of the 21-bp repeats. Additionally, CREB1 has also been found to primarily bind at the 5' LTR (rather than the 3' LTR) *in vivo *within HTLV-I infected cells, lending support to the idea that CREB1 is important for HTLV-I activated transcription [7].

If CREB1 is the dominant bZIP protein that is needed for Tax transactivation of the LTR, then what is the purpose of the additional bZIP proteins? Besides contributing to Tax transactivation, could these bZIP proteins help to exclude negative regulators from the LTR? A report by Basbous *et al. *[8] suggested that HBZ, which negatively down-regulated transcription from the HTLV-I LTR, heterodimerized with ATF-4 and subsequently this complex was no longer able to bind to the 21-bp repeats. Only over-expression of ATF-4 was found to reverse the negative effects of HBZ on Tax activity. However, additional studies are still needed to understand the respective contribution of CREB1 and other bZIP proteins, such as ATF-4, to Tax transactivation in the context of wildtype virus and stably integrated viral promoters (i.e. correctly assembled chromatinized DNA templates both *in vitro *and *in vivo*).

Lastly, Ching *et al. *presented the intriguing possibility of Tax enhancing transcription following transcription initiation. To determine whether Tax functioned solely to target TBP to the TATAA-element or if additional events subsequent to TBP (TFIID) recruitment were promoted by Tax, the authors constructed four independent reporters. Each promoter contained the minimal TATAA-element from HTLV-I, HIV-1, SV-40, or E1b promoters, two 21-bp repeats, and five copies of the Gal4-binding site. TBP was artificially targeted to the TATAA-element thru Gal4-TBP. The authors reasoned that if Tax functioned strictly to recruit TBP to the TATAA-element, then additional enhancement of transcription would not be observed when Tax and Gal4-TBP were present. Interestingly, only the Tax-responsive promoters, *i.e. *HTLV-I and HIV-1, were both synergistically stimulated by the addition of Tax and Gal4-TBP. These results suggest that Tax may control downstream transcription subsequent to the initiation phase.

Other viral transactivators have been shown to have a role at initiation and downstream events, such as elongation. The most notable of these has been Tat, the viral transactivator of HIV-1. Without cellular stimulation and Tat expression, RNAP II transcriptional elongation was shown to be inefficient, producing only short transcripts [9]. One major contributing factor of Tat-dependent transactivation is the elongation factor, pTEFb. pTEFb, composed of cyclin T1 and cdk9, associates with Tat leading to increased phosphorylation at specific sites on the heptad repeats of the CTD of RNAP II and promoting elongation. Elongation is highly dependent on the status of RNAP II CTD, since dissociation/association of factors have been shown to be dependent on CTD serine 5/serine 2 phosphorylation [1,10]. Hyperphosphorylation of CTD at serine 5 is associated with promoter clearance/early elongation, whereby initiation factors are released and the 5'capping machinery subsequently recruited. During processive elongation, there is a switch in CTD phosphorylation to serine 2 phosphorylation resulting in the loss of the capping machinery and the association of splicing, elongation and chromatin remodeling factors. In the case of HTLV-I, Tax has been shown not to associate with a CTD kinase [11] and a dominant negative mutant of cdk9 (the catalytic subunit of pTEFb) was found to increase Tax transactivation of the HTLV-I promoter [12]. Therefore, there is the possibility that other kinase complexes (small vs. large pTEFb complex or other cdk kinases) may aid in increased Tax transactivation. In this context, HTLV-I infected cells contain increased levels of cyclin E/cdk2 kinase activity, through sequestration of cdk inhibitor, p21/waf1, by cyclin D_2_/cdk4 complexes [13,14]. This kinase complex was able to phosphorylate RNAP II CTD and antibodies against cyclin E co-immunoprecipitated only the phosphorylated form of RNAP II from HTLV-I infected cells. Thus, if only indirectly, Tax may increase kinase activity resulting in enhanced CTD phosphorylation for steps following initiation, such as promoter clearance and/or elongation.

Processive elongation is highly dependent on remodeling of chromatin structure [1,10]. A study by Corey *et al. *[15] demonstrated that disruption of SWI/SNF recruitment by an activator resulted in lack of chromatin remodeling, transcription elongation, and production of full-length *hsp70 *mRNA. Tax has been shown to associate with BRG1 components of the ATP-dependent chromatin remodeling complex, SWI/SNF, and increase Tax transactivation [16]. Disruption of BRG1 by siRNA led to a decrease in Tax transactivation. Therefore, Tax may target SWI/SNF complexes downstream of RNAP II in order to prevent stalling of RNAP II. This raises a number of questions such as does Tax bind to an elongating RNAP II complex? Does Tax help to recruit elongation factors, such as TFIIS or TFIIF? Finally, it should be emphasized that each stage of transcription is not an independent process; coupling of the transcriptional and RNA processing machinery is thought to increase the rate and specificity of these enzymatic reactions [1]. As shown in Figure 1A, acetylation of nucleosomes and other transcription factors/coactivators promote an open complex structure and RNAP II holoenzyme assembly. Initiation by Tax is dependent on the recruitment of CBP/p300 and p/CAF by transcription factor/Tax complex at the 21-bp repeats (viral CRE elements). Phosphorylation of RNAP II CTD is important for loading of the 5' capping machinery to allow for rapid capping of nascent pre-mRNA, ensuring protection for the transcript from degradation. During promoter clearance (early elongation), site specific phosphorylation of the CTD is modified to allow for sequestration of splicing machinery and elongation factors, and release of the capping machinery. Assembly of SWI/SNF factors with Tax downstream of the elongation phase RNAP II complex remodels chromatin structure, promoting RNAP II processivity. Thus, the presence of Tax for initiation and possibly promoter clearance and/or elongation will help to increase viral transcription and mRNA processing overall (Figure 1B). While the results by Ching *et al. *are preliminary at this time, Tax transactivation post-initiation is indeed a novel concept. Further detailed analysis of Tax at both the LTR of HTLV-I and downstream of this region will help to resolve many of these questions and provide important insight into the transcription field.

**Figure 1:**
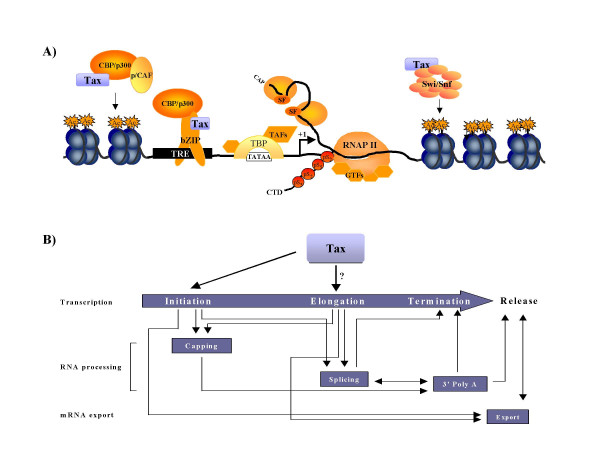
**Effect of Tax on transcription. **A) Schematic representation of proximal promoter of HTLV-I. Tax binding to CBP/p300 with either p/CAF or bZIP transcription factors (*e.g. *CREB1) leads to increased acetylation and interaction with the basal transcription machinery. Tax binding to SWI/SNF downstream of start site may help to remodel restrictive chromatin structure and aid in promoter clearance and elongation. B) The possible effect of Tax on gene expression network. The sequential steps of transcription (initiation, elongation, and termination) are intricately linked together and to mRNA processing and export (adapted from ref. 1). Thus, the effect of Tax on initiation and possibly elongation (both early promoter clearance and processive elongation events) would contribute, albeit indirectly, to RNA processing and export.

## Abbreviations

HTLV-I, human T cell leukemia virus, type I

CRE, cAMP response element

CREB, cAMP response element binding protein

ChIP, chromatin immunoprecipitation

RNAP II, RNA polymerase II

CTD, C-terminal domain

HIV-1, human immunodeficiency virus, type 1

LTR, long terminal repeat

TBP, TATA binding protein

TxREs, Tax-responsive elements

GTFs, general transcription factors

TAR, transactivation region

## Competing Interests

None declared.

## Authors' contributions

Both authors contributed equally to the structure and content of the manuscript.
